# Association between resilience promotion factors during childhood and risk of drug use disorder during adulthood

**DOI:** 10.46439/addiction.2.007

**Published:** 2023

**Authors:** Ann Aschengrau, Michael R. Winter, Margaret G. Shea

**Affiliations:** 1 Department of Epidemiology, Boston University School of Public Health, 715 Albany Street, Boston, MA 02118, USA; 2 Biostatistics and Epidemiology Data Analytics Center, Boston University School of Public Health, 85 East Newton Street, Boston MA 02118, USA

**Keywords:** Resilience, Drug use disorder, Adverse childhood experiences

## Abstract

Few studies have been conducted on the relationship between “outside-residing” resilience characteristics and the risk of developing drug use disorder later in life. These characteristics include responsive and caring parenting, household routines involving regular family meals and bedtime routines, social support from peers, participation in organized activities, and religious service attendance. We quantified the association between these resilience promotion factors during childhood and the risk of developing criteria for drug use disorder during adulthood using data from a retrospective cohort study of 618 adults born in Massachusetts during 1969–1983, including those with adverse childhood experiences (ACEs). Self-administered questionnaires gathered information on criteria for drug use disorder, ACEs, and family and community resilience promotion factors. Compared to individuals with “low” numbers of resilience promotion factors, 30% (95% CI: 0.5–0.9) and 50% reductions (95% CI: 0.4–0.8) in the risk of developing one or more criteria for drug use disorder were observed among those with “moderate” and “high” numbers of resilience factors, respectively (p value for trend=0.003). Overall, family factors were associated with greater risk reductions than comparable numbers of community factors. Among individuals with ACEs, a “high” number of family factors but not community factors were associated with a reduction in risk (RR:0.6, 95% CI:0.4–1.0 for family factors, RR:1.0, 95% CI:0.5–1.8 for community factors). These results suggest that the risk of developing criteria for drug use disorder decreases in a dose-response fashion according to the number of “outside-residing” resilience promotion factors during childhood, and that family factors are associated with greater risk reductions than community factors, particularly among individuals with ACEs. Coordinated prevention efforts at the family and community level are recommended to reduce the risk of this important societal problem.

## Introduction

Drug use disorder is a major societal problem characterized by clinically significant impairments, including physical and emotional health problems, persistent or increasing drug use and failure to meet major responsibilities at home, school and work [1]. According to the latest National Survey on Drug Use and Health, an estimated 18.4 million people in the U.S. population had a drug use disorder in the past year [1]. Despite its substantial consequences and high prevalence, much remains to be learned about the complex interplay of factors that impact its development.

The well-known biopsychosocial framework is a useful model for describing the complex interactions over the life course that contribute to drug use and drug use disorder [2]. According to this model, the decision to engage in or refrain from drug use and develop drug use disorder is shaped not only by biological factors such genetic vulnerability and psychological characteristics such as self-esteem and personality type, but also by social factors such as family dynamics and personal experiences. This multi-factorial framework includes factors that promote “resilience,” which can be defined as “positive adaptation or the ability to maintain or regain mental health despite experiencing adversity” [3]. Resilience promotion factors comprise a wide range of factors including those residing within individuals, such as genetics, natural temperament, knowledge and skills, and those residing outside of individuals, such as social supports and cultural and societal resources [4].

Available evidence suggests that the risk of drug use is reduced among individuals whose childhood was characterized “outside-residing” resilience factors such as a positive home environment [5,6], positive student-teacher relationships [7], organized activities [8,9], and religious service attendance [10,11]; however, none of these studies examined their impact on the risk of developing drug use disorder.

Prior studies have also found that ACEs increase the subsequent risk of drug use (e.g., [7,12–15]) and drug use disorder [16–19]. However, only a few studies have examined the interplay of resilience promotion factors and ACEs on the risk of drug use in the same population (e.g., [7,14,19]). Furthermore, to the best of our knowledge, only two studies have examined whether resilience promotion factors reduced the risk of drug use disorder among individuals with ACEs. A longitudinal study by LeTendre and Reed did not find that attendance at religious services and activities reduced the risk of drug use disorder among individuals with ACEs [19]. However, a cross-sectional study by Wingo et al. found that high levels of core resilience were associated with a small reduction in the risk of drug use disorder after taking into account a history of child abuse [14].

Because there is a great need to identify modifiable measures for developing criteria for drug use disorder, we undertook the present analysis in a population-based cohort study to quantify the associations between eleven “outside-residing” resilience promotion factors during childhood and developing criteria for drug use disorder, particularly among individuals with ACEs. We hypothesized that the risk of developing criteria for drug use disorder would decrease as the number of resilience promotion factors increased, that family factors would have a greater impact than community factors and that the decreased risks would be attenuated among individuals with ACEs.

## Materials and Methods

### Selection of study population

The present analysis is based on data from the Cape Cod Health Study (CCHS), a retrospective cohort study of individuals born in the Cape Cod area of Massachusetts. Cape Cod is a popular summer tourist area along the Massachusetts coastline comprised of suburban towns with mainly white, non-Hispanic residents. The goal of the CCHS was to examine the individual and combined impacts of early life exposure to tetrachloroethylene (PCE)-contaminated drinking water and social factors on risky behaviors, including drug use and drug use disorder. PCE is an organic solvent whose potential to cause neurotoxic effects has been established through numerous animal and human studies [20,21].

Individuals were eligible for the study if they were born from 1969 through 1983 to married women living in Cape Cod towns known to have some neighborhoods with PCE-contaminated drinking water. Potential participants were identified by reviewing approximately 13,000 birth records to identify exposed individuals (N=1,910). This was accomplished by cross-matching the maternal address on the record with information from the local water companies on the locations affected by the contamination. Unexposed individuals were randomly selected from the remaining individuals after frequency matching to exposed individuals on month and year of birth (N=1,928). Another 1,202 older siblings of exposed and unexposed individuals born in Massachusetts during 1969–1983 were identified for a total of 5,040 selected individuals. Birth records of all individuals were reviewed to obtain information on the family, including the names of the individuals and their parents; the individual’s date of birth, birth weight and gestational duration; and the parents’ ages, mother’s educational level and father’s occupation when the individual was born.

### Follow-Up and Enrollment

During Phase 1 of the study (2000–2005), the selected 5,040 individuals were traced and sent invitation letters describing the purpose of the study and requesting that they complete a self-administered questionnaire. Overall, 40.5% of successfully located individuals returned the study questionnaire (N=1,689). The Phase 1 questionnaire gathered information on cigarette smoking, alcoholic beverage consumption, and drug use, demographic and medical characteristics, and residential addresses.

Phase 2 of the study (2017–2020) focused on the 1,512 Phase 1 respondents with adequate residential data. A total of 694 participants completed the entire Phase 2 survey (46.8%). The remainder never responded to several contact attempts (N=718), were not found (N=54), were deceased (N=6), declined to participate (N=26), or completed only a small portion of the survey (N=14). Comparison of Phase 2 participants and non-participants found that they were similar on many characteristics including PCE exposure status, age, race, maternal age, birth weight, gestational age, and receipt of prenatal care. However, compared to Phase 2 non-participants, a larger proportion of Phase 2 participants were female, had mothers who were college graduates and fathers employed in white collar jobs, and a smaller proportion ever smoked cigarettes regularly, reported a recent heavy drinking episode and ever used drugs.

The Phase 2 questionnaire updated information on demographic, medical, and behavioral characteristics. The questionnaire also collected information on the eleven established Diagnostic and Statistical Manual of Mental Disorders Fifth Edition (DSM5) criteria for drug use disorder for any drug. Questions for these criteria, which were adapted from the validated Alcohol Use Disorder and Associated Disabilities Interview Schedule (AUDADIS) (National Institute of Alcohol Abuse and Alcoholism), ascertained the lifetime prevalence of the following behaviors: ever engaging in hazardous use that increased the chances of getting hurt, ever having social/interpersonal problems due to use, often neglecting major roles due to use, ever developing tolerance such that the usual drug amount had much less of an effect than it once did, often using larger amounts or for a longer period of time than intended, ever making unsuccessful attempts to stop or cut down use, ever spending much time getting or using the drug, ever continuing to use a drug despite having physical and psychological problems related to use, ever giving up activities due to use, ever developing cravings for the drug, and ever having withdrawal symptoms when the drug effects were wearing off.

We also developed eleven questionnaire items on potentially modifiable “outside-residing” resilience promotion factors during childhood that available evidence suggested may reduce the development of risk-taking behaviors. These include responsive and caring parenting; household routines involving regular family meals and bedtime routines; social support from peers, participation in organized activities, and religious service attendance [5–11,19].

Lastly, an updated version of the Adverse Childhood Experiences (ACE) Study questionnaire developed by Felliti et al. [23] was adapted to assess adverse experiences before the age of 18 years [24]. In particular, information was gathered on the occurrence of emotional, physical, and sexual abuse; physical and emotional neglect; household substance abuse and mental illness; parental arguing, separation, and divorce; and exposure to community violence. The ACE questionnaire has good to excellent reliability [25]. The entire study questionnaire was developed and pre-tested in conjunction with survey research experts from the University of Massachusetts Center for Survey Research. Informed consent was obtained from all participants.

### Statistical analysis

First, we compared the reported number and type of resilience promotion factors overall and according to ACE history. Second, we examined the distribution of participant characteristics according to the reported number of resilience promotion factors. Next, we compared the criteria for drug use disorder among participants in relation to the reported number resilience promotion factors during childhood. In particular, we examined the lifetime presence of any criteria (>=1) of drug use disorder and groupings of the number of criteria (1–2, 3–11) in relation to the overall number of resilience promotion characteristics during childhood (2–7, 8–9, 10–11), the number of family resilience factors (0–3, 4–5, 6) and the number of community factors (1–2, 3–4, 5), with each category corresponding to “low,” “moderate,” and “high numbers of factors. The frequency of specific resilience promotion factors (e.g., participation in organized activities) was too high to examine them individually. Categories of the resilience promotion factors were defined by examining their frequency distributions without regard to outcome status. These analyses were conducted in the entire sample and repeated among individuals with adverse childhood experiences.

Risk ratios (RR) were used to estimate the strength of the associations between resilience promotion characteristics and criteria for drug use disorder. Ninety-five percent confidence intervals were used to assess the precision of the RRs. Tests for trend were used to examine dose-response relationships between the number of resilience promotion factors and criteria for drug use disorder. First, crude analyses were conducted and then generalized estimating equation (GEE) analyses were performed to account for non-independent outcomes arising from several children from the same family [26,27]. Twelve percent of Phase 2 participants were siblings. Lastly, adjusted GEE analyses were conducted to control for confounding variables. Covariates considered for these analyses were factors associated with drug use disorder criteria that, according to our directed acyclic graph (DAG), also influenced the resilience promotion factors. These variables included the participant’s sex and age at questionnaire completion (a proxy for year of birth); mother’s age and educational level, father’s occupation and median income level in the town of residence when the participant was born. The latter measure of community socioeconomic status was obtained from U.S. Census data. Early life exposure to PCE-contaminated drinking water was not controlled since it did not differ according to the number of resilience factors (Table 2).

## Results

A large proportion of the study population experienced high numbers of resilience promotion factors during childhood (Table 1). In fact, all participants reported at least two resilience promotion factors and 79.8% of participants reported eight or more factors. The most commonly reported resilience promotion factors included having at least one good friend in elementary school (98.4%) and high school (98.5%); having affectionate and caring parental figures (91.8%); and having regular bedtime routines (86.6%) and frequent family dinners (91.9%) in elementary school.

While resilience factors were also common among individuals with ACEs, their frequencies tended to be lower than individuals without ACEs (Table 1). For example, the proportions with high numbers (10–11) of resilience factors was 34.9% among participants with ACEs vs. 50.8% among those without ACEs.

Many characteristics of participants and their parents were similar among those with “moderate to high” (8–11) and “low” numbers (2–7) of resilience promotion factors during childhood (Table 2). For example, the groups were comparable with respect to race; maternal receipt of prenatal care, obstetrical or medical complications and alcoholic beverage consumption during gestation; number of older siblings, median family income in their birth communities, and early life exposure to PCE-contaminated drinking water (Table 2). However, compared to participants with “low” numbers (2–7) of resilience promotion factors, participants with “moderate to high” numbers (8–11) of factors were slightly younger, and more likely to be male, have older parents, more educated mothers, and fathers in white collar occupations. Participants with “high” numbers of factors were also more likely to be college graduates, less likely to be separated, divorced or widowed, and less likely to have mental disorders.

Overall, 23.3% of participants reported having at least one criterion for drug use disorder over their lifetime. Compared to participants with a “moderate” to “high” number of resilience promotion factors, individual criteria for drug use disorder were reported 1.6 to 2.7 times more frequently among participants with a “low” number of resilience factors (Table 3). Criteria that were most common among participants with a “low” number of resilience factors included social and interpersonal problems related to use, developing a tolerance for drugs, and neglecting major roles due to use.

The presence of resilience promotion factors during childhood was associated with a reduced risk of having at least one criterion for drug use disorder. Compared to participants with a “low” number (2–7) of resilience promotion factors, participants with a “moderate” number of factors (8–9) had a 30% reduced risk of having any criteria for drug use disorder (adjusted RR: 0.7, 95% CI: 0.5–0.9) and those with a “high” number (10–11) of factors had a 50% reduced risk (adjusted RR: 0.5, 95% CI: 0.4–0.8, p value for trend=0.003) (Table 4). The inverse association was slightly stronger among participants with more severe drug use disorder. In particular, participants with 8–9 resilience factors had a 40% reduced risk of having three to eleven criteria for drug use disorder (adjusted RR: 0.6, 95% CI: 0.3–1.0) while participants with 10–11 resilience factors had a 60% reduced risk (adjusted RR 0.4; 95% CI: 0.2–0.8, p value for trend = 0.006).

Higher numbers of both family and community factors were associated with reductions in the risk of developing any criteria for drug use disorder; however, higher numbers of family factors were associated with slightly larger reductions in risk (Table 4). For example, the highest number of family factors (N=6) was associated with a 50% risk reduction (adjusted RR: 0.5, 95% CI: 0.3–0.7) whereas the highest number of community factors (N=5) was associated with a 30% risk reduction (adjusted RR: 0.7, 95% CI: 0.4–1.2). This trend held for participants with more severe drug use disorder.

While the overall number of reported resilience factors was associated with 30% reductions in the risk of developing criteria for drug use disorder among participants with ACEs (adjusted RR: 0.7, 95% CI: 0.5–1.0 for 10–11 and 8–9 factors), the risk reduction stemmed from the family factors (Table 5). For example, the highest number of family factors was associated with a 40% risk reduction (adjusted RR: 0.6, 95% CI: 0.4–1.0) whereas the highest number of community factors was not associated with any reduction in risk (adjusted RR: 1.0, 95% CI: 0.5–1.8).

## Discussion

The results of this study suggest that the risk of developing criteria for drug use disorder during adulthood decreases in a dose-response fashion according to the number of “outside-residing” resilience promotion factors during childhood and that a high number of family factors is associated with greater risk reductions than a high number of community factors. However, only a high number of family factors was associated with risk reductions among individuals with ACEs.

Several limitations of the study should be considered when interpreting these results. The first is exposure misclassification due to inaccurate reporting of resilience promotion factors and ACEs resulting in bias towards the null. Participants were on average 40–41 years old when they competed the study questionnaire about their childhood experiences. Thus, it is quite possible that their memories were inaccurate due to the long recall period. It is also possible that their reports of resilience promotion factors and ACEs were inaccurate for other reasons, such as the desire to give socially desirable responses and, repressed memories in the case of adverse experiences. Unfortunately, our only source of information about these experiences were self-reports and so their accuracy could not be verified. Furthermore, the frequency of specific resilience factors was too high to be examined individually.

Another limitation of this study stems from the use of self-reports as the source of information on the criteria for drug use disorder rather than diagnoses by clinicians trained in addiction medicine. Based on this information, the lifetime prevalence of developing at least one and two or more criteria for drug use disorder in our study population were 23.3% and 14.4%, respectively. This is higher than the 9.9% lifetime prevalence of drug use disorder (based on two or more criteria) observed in a nationally representative survey of U.S. adults [28]. While estimates of the lifetime frequency of drug use disorder are not available for Cape Cod residents, these figures are not out of line with the number of opioid overdose deaths among Cape Cod residents during the study period (Massachusetts Department of Public Health. Registry of Vital Records and Statistics. Opioid Overdose Death Rates, All Intents, by County, Massachusetts Residents, 2010–2020. (https://www.mass.gov/doc/opioid-related-overdose-deaths-by-county-may-2021/download Accessed April 2023).

The final study limitation stems from the low questionnaire response rate. Although this problem reduced the statistical power of the study, the following evidence suggests that it did not bias the observed associations *away* from the null. First, potential study participants were not told the specific hypotheses under investigation when they were invited to take part in the study. Second, losses stemming from the death of potential participants were relatively small (N=117) and our review of death records from the Massachusetts Registry of Vital Records and Statistics and the National Death Index found that only seven of these deaths were associated with substance use. Third, many available characteristics of participants and non-participants were similar, including age, race, and maternal age when the participant was born. However, participants were more likely than non-participants to come from families with higher socioeconomic status and less likely to report drug use. Thus, it is likely that the frequency of drug use disorder is higher and the frequency of resilience promotion factors is lower among non-participants. The likely impact of these selection factors was to bias the results towards the null and so the true associations may be stronger than those observed.

The results are in agreement with the literature on the benefits of resilience promotion factors in reducing the risk of drug use. Thus far, this literature suggests that a positive family environment [5], peer support [6], positive student-teacher relationships [7], organized activities [8,9], and religious service attendance [10,11] reduce the risk of drug use.

To date, only two prior studies have examined the impact of resilience promotion factors on drug use disorder [14,19]. The longitudinal study by LeTendre and Reed [19] found that attendance at religious services and activities reduced the risk of developing drug use disorder but no moderating impacts were observed among individuals with ACEs (Odds Ratio: 1.0 for interaction between adverse childhood experiences and religiosity, 95% CI:0.0.95–1.06). In contrast, the cross-sectional study by Wingo et al. [14] found that higher resilience as measured by the Connor-Davidson Resilience Scale was associated with a 4% reduction in the lifetime risk drug use disorder after controlling for child abuse (Odds Ratio: 0.96, 95% CI:0.94–0.98). These two sets of results are difficult to compare because they examined different sources of resilience: Letendre and Reed’s study focused on one source of “outside-residing” resilience while Wingo et al. examined “core” resilience characteristics such as tenacity, emotional control under pressure and goal orientation. The results of the two prior studies as well as our current study suggest that future research would benefit from examining the impacts of multiple sources of both “outside-residing” and “core” resilience factors.

There are many possible psychosocial mechanisms by which “outside-residing” resilience promotion factors reduce the risk of drug use disorder. Both family- and community factors help build emotional stability, increase self-confidence and improve social skills (e.g., [29]). They also reduce feelings of isolation, alienation, psychopathology such as depression and anxiety (e.g., [14,30]). Lastly, these factors are also important sources of adult and peer role models who can facilitate positive development and promote social norms that may include negative attitudes towards drug use (e.g., [31]). The biological mechanisms by which these beneficial factors may influence an individual’s risk of drug use and drug use disorder are currently unknown. However, a growing literature suggests that the biological basis for resilience involves changes in neuronal activity in the medial and dorsolateral prefrontal cortex, hippocampus, ventral tegmental area and corticostriatal connectivity levels (e.g., [32–34]) and protective effects of oxytocin in counterbalancing the cortisol stress response [35].

## Conclusions

The results of this study suggest that the risk of developing criteria for drug use disorder during adulthood decreases in a dose-response fashion according to the number of “outside-residing” resilience promotion factors during childhood and that, in general, family factors are associated with greater risk reductions than community factors. They also suggest that only high numbers of family factors are associated with risk reductions among individuals with ACEs. Given the paucity of the literature, we suggest that further research on the potentially beneficial effects of resilience promotion factors on the risk of drug use and drug use disorder be conducted, especially among individuals with ACEs. This research would be most informative if both “core” and “outside-residing” factors were examined individually in a prospective cohort study beginning in childhood. Lastly, because the current study population was comprised of the offspring of married couples who were primarily Whites living in suburban areas, future studies would benefit greatly from including racially, ethnically, and socioeconomically diverse participants.

In the meantime, these findings imply that comprehensive and coordinated prevention efforts by healthcare and social service providers, schools and religious organizations in close collaboration with families may be required to reduce the risk of drug use disorder. Recognizing that childhood resilience factors and ACEs impact an individual’s risk-taking behaviors later in life will help these professionals as they work closely with families to shape effective and acceptable risk reduction approaches.

## Figures and Tables

**Table 1.** Frequency of Reported “Outside-Residing” Resilience Promotion Factors by ACE History, Cape Cod Health Study.

	All Participants (N=618)	Participants with Any ACEs (N=618)	Participants without Any ACEs (N=185)
*Overall Number of Reported “Outside-Residing” Resilience Factors*			
10–11 (“High”)	245 (39.6)	151 (34.9)	94 (50.8)
8–9 (“Moderate”)	248 (40.1)	170 (39.3)	78 (42.2)
2–7 (“Low”)	125 (20.2)	112 (25.9)	13 (7.0)
*Number of Reported Family Factors*			
6 (“High”)	267 (43.2)	158(36.5)	109 (58.9)
4–5 (“Moderate”)	274 (44.3)	201 (46.4)	73 (39.5)
0–3 (“Low”)	77 (12.5)	74 (17.1)	3 (1.6)
*Number of Reported Community Factors*			
5 (“High”)	247 (40.0)	158 (36.5)	89 (48.1)
3–4 (“Moderate”)	330 (53.4)	240 (55.4)	90 (48.6)
1–2 (“Low”)	41 (6.6)	35 (8.1)	6 (3.2)
*Type of Resilience Factor*			
Affectionate and caring parental figures	567 (91.8)	385 (88.9)	182 (98.4)
Regular bedtime routine in elementary school*	535 (86.6)	363 (83.8)	172 (93.0)
Regular bedtime in elementary school*	593 (96.0)	412 (95.2)	181 (97.8)
Regular bedtime in high school*	363 (58.7)	235 (54.3)	128 (69.2)
Frequent* family dinners in elementary school	568 (91.9)	386 (89.2)	182 (98.4)
Frequent* family dinners in high school	426 (68.9)	269 (62.1)	157 (84.9)
At least one good friend in elementary school	608 (98.4)	424 (97.9)	184 (99.5)
At least one good friend in high school	609 (98.5)	424 (97.9)	185 (100)
Frequent** religious service attendance in elementary school	386 (62.5)	258 (59.6)	128 (69.2)
Frequent** religious service attendance in high school	285 (46.1)	187 (43.2)	98 (53.0)
Participated in organized activities in high school	529 (85.6)	360 (83.1)	169 (91.4)

* Often or always

** At least once a month

**Table 2.** Distribution of Selected Characteristics by Reported “Outside-Residing” Resilience Promotion Factors during Childhood, Cape Cod Health Study.

Characteristic	“Moderate to High” Number (8–11) of Resilience Promotion Factors (N=493)		“Low” Number (2–7) of Resilience Promotion Factors (N=125)	
	n	%	n	%
Year of birth				
1969–1974	111	22.5%	32	25.6%
1975–1980	257	52.1%	68	54.4%
1981–1983	125	25.4%	25	20.0%
Current age (median)	40		41	
Sex at Birth				
Male	180	36.5%	33	26.4%
Female	313	63.5%	92	73.6%
% White race	487	98.8%	125	100%
Current Educational Level				
High school graduate or less	20	4.1%	14	11.2%
Some college	78	15.9%	32	25.6%
Fourth year college grad or higher	393	80.0%	79	63.2%
Missing	2		0	
Current marital status				
Married or cohabitating	415	84.2%	99	79.2%
Separated, divorced, widowed	29	5.9%	13	10.4%
Never married	49	9.9%	13	10.4%
Mental disorder*				
Yes	182	37.1%	61	49.6%
No	308	62.9%	62	50.4%
Missing	3		2	
Mother’s age at participant’s birth (median)	28		26	
Father’s age at participant’s birth (median)	30		28	
Mother’s educational level at participant’s birth				
High school graduate or less	142	28.9%	53	42.4%
Some college	153	31.1%	39	31.2%
Fourth year college grad or higher	197	40.0%	33	26.4%
Missing	1		0	
Father’s occupation at participant’s birth				
White collar	277	56.5%	56	45.5%
Blue collar	138	28.2%	45	36.6%
Other	75	15.3%	22	17.9%
Missing	3		2	
Mother received prenatal care during participant’s gestation				
Yes	478	99.8%	117	100.0%
No	1	0.2%	0	0.0%
Missing	14		8	
Mother smoked cigarettes during participant’s gestation				
Yes	92	21.6%	26	28.6%
No	334	78.4%	65	71.4%
Missing	67		34	
Mother consumed alcohol during participant’s gestation				
Yes	200	47.1%	41	45.1%
No	225	52.9%	50	54.9%
Missing	68		34	
Mother had medical and obstetrical complications during participant’s gestation				
Yes	92	21.7%	19	21.3%
No	331	78.3%	70	78.7%
Missing	70		36	
Early life exposure to PCE contaminated drinking water	293	59.4%	70	56.0%
Number of older siblings				
0	229	46.5%	60	48.4%
1+	263	53.4%	64	51.6%
Missing	1		1	
Birth Community Median Family Income According to U.S. Census (median)	30,468		30,468	

* Mental disorder includes depression, anxiety, PTSD, bipolar disorder and eating disorder.

**Table 3.** Specific Criteria for Drug Use Disorder According to Reported “Outside-Residing” Resilience Promotion Factors during Childhood, Cape Cod Health Study

Criteria for Drug Use Disorder	“Low” Number (2–7) of Resilience Promotion Factors N=493		“Moderate” to “High” Number (8–11) of Resilience Promotion Factors N=125	
	n	%	n	%
Hazardous use	24	19.2	42	8.5
Social/interpersonal problems related to use	12	9.6	17	3.5
Often neglected major roles to use	11	8.8	18	3.7
Experienced withdrawal symptoms	17	13.6	33	6.7
Developed tolerance	22	17.6	32	6.5
Used larger amounts or for longer time than intended	17	13.6	33	6.7
Made unsuccessful attempts to quit or control use	21	16.8	50	10.1
Spent much time getting or using drug	17	13.6	42	8.5
Physical/psychological problems related to use	14	11.2	27	5.5
Gave up activities given up due to use	6	4.8	13	2.6
Developed craving	19	15.2	45	9.1

**Table 4.** Lifetime Criteria of Drug Use Disorder in Relation to “Outside-Residing” Resilience Promotion Factors During Childhood, Cape Cod Health Study.

Outcome	Resilience Factor History	% Yes(n/N)	Crude RR (95% Cl)	Adjusted* GEE RR (95% Cl)	P value for trend
*Overall Number of Reported Factors*					
Any (>=1) Criteria of Drug Use Disorder	10–11 Factors	18.8 (46/245)	0.5 (0.4–0.8)	0.5 (0.4–0.8)	0.003
	8–9 Factors	22.2 (55/248)	0.6 (0.5–0.9)	0.7 (0.5–0.9)	
	2–7 Factors	34.4 (43/125)	Reference	Reference	
One-Two Criteria of Drug Use Disorder	10–11 Factors	11.6 (26/225)	0.6 (0.3–1.0)	0.6 (0.3–1.0)	0.10
	8–9 Factors	12.3 (27/220)	0.6 (0.4–1.1)	0.6 (0.4–1.1)	
	2–7 Factors	19.6 (20/102)	Reference	Reference	
Three-Eleven Criteria of Drug Use Disorder	10–11 Factors	9.1 (20/219)	0.4 (0.2–0.7)	0.4 (0.2–0.8)	0.006
	8–9 Factors	12.7 (28/221)	0.6 (0.4–0.95)	0.6 (0.3–1.0)	
	2–7 Factors	21.9 (23/105)	Reference	Reference	
*Number of Reported Family Factors*					
Any (>=1) Criteria of Drug Use Disorder	6 Factors	15.7 (42/267)	0.4 (0.3–0.7)	0.5 (0.3–0.7)	<0.001
	4–5 Factors	27.4 (75/274)	0.8 (0.5–1.1)	0.7 (0.5–1.1)	
	0–3 Factors	35.1 (27/77)	Reference	Reference	
One-Two Criteria of Drug Use Disorder	6 Factors	8.9 (22/247)	0.4 (0.2–0.7)	0.4 (0.2–0.7)	0.002
	4–5 Factors	15.3 (36/235)	0.7 (0.4–1.1)	0.6 (0.3–1.1)	
	0–3 Factors	23.1 (15/65)	Reference	Reference	
Three-Eleven Criteria of Drug Use Disorder	6 Factors	8.2 (20/245)	0.4 (0.2–0.8)	0.4 (0.2–0.9)	0.009
	4–5 Factors	16.4 (39/238)	0.8 (0.5–1.5)	0.8 (0.4–1.6)	
	0–3 Factors	19.4 (12/62)	Reference	Reference	
*Number of Reported Community Factors*					
Any (>=1) Criteria of Drug Use Disorder	5 Factors	19.4 (48/247)	0.7 (0.4–1.3)	0.7 (0.4–1.2)	0.175
	3–4 Factors	25.8 (85/330)	1.0 (0.6–1.6)	0.8 (0.5–1.4)	
	1–2 Factors	26.8 (11/41)	Reference	Reference	
One-Two Criteria of Drug Use Disorder	5 Factors	11.9 (27/226)	0.8 (0.3–2.0)	0.8 (0.3–1.8)	0.713
	3–4 Factors	14.3 (41/286)	1.0 (0.4–2.4)	0.8 (0.3–1.9)	
	1–2 Factors	14.3 (5/35)	Reference	Reference	
Three-Eleven Criteria of Drug Use Disorder	5 Factors	9.5 (21/220)	0.6 (0.2–1.3)	0.6 (0.2–1.4)	0.102
	3–4 Factors	15.2 (44/289)	0.9 (0.4–2.0)	0.8 (0.4–2.0)	
	1–2 Factors	16.7 (6/36)	Reference	Reference	

* Adjusted for: age, sex, maternal age, maternal education, paternal occupation, median adjusted family income

**Table 5.** Any (≥1) Lifetime Criteria of Drug Use Disorder in Relation to “Outside-Residing” Resilience Promotion Factors among Participants with History of ACEs, Cape Cod Health Study.

	% Yes(n/N)	Crude RR (95% Cl)	Adjusted* GEE RR (95% Cl)	P value for trend
*Overall Number of Reported Resilience Factors*				
10–11 Factors	26.5 (40/151)	0.7 (0.5–1.0)	0.7 (0.5–1.0)	0.085
8–9 Factors	27.1 (46/170)	0.7 (0.5–1.0)	0.7 (0.5–1.0)	
2–7 Factors	36.6 (41/112)	Reference	Reference	
*Number of Reported Family Factors*				
6 Factors	21.5 (34/158)	0.6 (0.4–0.9)	0.6 (0.4–1.0)	0.043
4–5 Factors	33.3 (67/201)	0.9 (0.7–1.4)	0.9 (0.6–1.4)	
0–3 Factors	35.1 (26/74)	Reference	Reference	
*Number of Reported Community Factors*				
5 Factors	25.9 (41/158)	1.0 (0.5–1.9)	1.0 (0.5–1.8)	0.56
3–4 Factors	32.1 (77/240)	1.2 (0.7–2.3)	1.1 (0.6–2.0)	
1–2 Factors	25.7 (9/35)	Reference	Reference	

* Adjusted for: age, sex, maternal age, maternal education, paternal occupation, median adjusted family income

## References

[1] Substance Abuse and Mental Health Services Administration, 2021. Key Substance Use and Mental Health Indicators in the United States; Results from the 2020 National Survey on Drug Use and Health, HHS Publication No. PEP21-07-01-003, NSDUH Series H-56). Rockville, MD: Center for Behavioral Health Statistics and Quality, Substance Abuse and Mental Health Services Administration. Retrieved from https://www.samhsa.gov/data/, July 15, 2022.

[2] Engel GL (1992). The need for a new medical model: A challenge for biomedicine. Family Systems Medicine, 10(3), 317–331.

[3] Herrman H, Stewart DE, Diaz-Granados N, Berger EL, Jackson B, Yuen T. What is resilience?. The Canadian Journal of Psychiatry. 2011 May;56(5):258–65. 21586191 10.1177/070674371105600504

[4] Zimmerman MA. Resiliency theory: A strengths-based approach to research and practice for adolescent health. Health Education & Behavior. 2013 Aug;40(4):381–3. 23863911 10.1177/1090198113493782 PMC3966565

[5] Martin MJ, Conger RD, Sitnick SL, Masarik AS, Forbes EE. Reducing risk for substance use by economically disadvantaged young men: Positive family environments and pathways to educational attainment. Child Development. 2015 Nov;86(6):1719–37. 26307026 10.1111/cdev.12413 PMC4626283

[6] Hodder RK, Freund M, Bowman J, Wolfenden L, Gillham K. Association between adolescent tobacco, alcohol and illicit drug use and individual and environmental resilience protective factors. BMJ open. 2016 Nov 1;6(11):e012688. 10.1136/bmjopen-2016-012688 PMC5168489 27888175

[7] Forster M, Gower AL, Borowsky IW, McMorris BJ. Associations between adverse childhood experiences, student-teacher relationships, and non-medical use of prescription medications among adolescents. Addictive behaviors. 2017 May 1;68:30–4. 28088740 10.1016/j.addbeh.2017.01.004

[8] Driessens CM. Extracurricular activity participation moderates impact of family and school factors on adolescents’ disruptive behavioural problems. BMC Public Health. 2015 Dec;15(1):1–3. 26558510 10.1186/s12889-015-2464-0 PMC4642774

[9] McCabe KO, Modecki KL, Barber BL. Participation in organized activities protects against adolescents’ risky substance use, even beyond development in conscientiousness. Journal of Youth and Adolescence. 2016 Nov;45:2292–306. 26979446 10.1007/s10964-016-0454-x

[10] Rasic D, Kisely S, Langille DB. Protective associations of importance of religion and frequency of service attendance with depression risk, suicidal behaviours and substance use in adolescents in Nova Scotia, Canada. Journal of Affective Disorders. 2011 Aug 1;132(3):389–95. 21458077 10.1016/j.jad.2011.03.007

[11] Chen Y, VanderWeele TJ. Associations of religious upbringing with subsequent health and well-being from adolescence to young adulthood: An outcome-wide analysis. American Journal of Epidemiology. 2018 Nov 1;187(11):2355–64. 30215663 10.1093/aje/kwy142 PMC6211237

[12] Dube SR, Felitti VJ, Dong M, Chapman DP, Giles WH. Childhood abuse, neglect, and household dysfunction and the risk of illicit drug use: the adverse childhood experiences study. Pediatrics. 2003 Mar;111(3):564–72. 12612237 10.1542/peds.111.3.564

[13] Mersky JP, Topitzes J, Reynolds AJ. Impacts of adverse childhood experiences on health, mental health, and substance use in early adulthood: A cohort study of an urban, minority sample in the US. Child Abuse & Neglect. 2013 Nov 1;37(11):917–25. 23978575 10.1016/j.chiabu.2013.07.011 PMC4090696

[14] Wingo AP, Ressler KJ, Bradley B. Resilience characteristics mitigate tendency for harmful alcohol and illicit drug use in adults with a history of childhood abuse: A cross-sectional study of 2024 inner-city men and women. Journal of psychiatric research. 2014 Apr 1;51:93–9. 24485848 10.1016/j.jpsychires.2014.01.007 PMC4605671

[15] Forster M, Grigsby TJ, Rogers CJ, Benjamin SM. The relationship between family-based adverse childhood experiences and substance use behaviors among a diverse sample of college students. Addictive behaviors. 2018 Jan 1;76:298–304. 28889058 10.1016/j.addbeh.2017.08.037

[16] Myers B, McLaughlin KA, Wang S, Blanco C, Stein DJ. Associations between childhood adversity, adult stressful life events, and past-year drug use disorders in the National Epidemiological Study of Alcohol and Related Conditions (NESARC). Psychology of Addictive Behaviors. 2014 Dec;28(4):1117–26. 25134042 10.1037/a0037459 PMC4274198

[17] Stein MD, Conti MT, Kenney S, Anderson BJ, Flori JN. Adverse childhood experience effects on opioid use initiation, injection drug use, and overdose among persons with opioid use disorder. Drug and Alcohol Dependence. 2017 Oct 1;179:325–9. 28841495 10.1016/j.drugalcdep.2017.07.007 PMC5599365

[18] Wolitzky-Taylor K, Sewart A, Vrshek-Schallhorn S, Zinbarg R, Mineka S. The effects of childhood and adolescent adversity on substance use disorders and poor health in early adulthood. Journal of Youth and Adolescence. 2017 Jan;46:15–27. 27613006 10.1007/s10964-016-0566-3 PMC5219858

[19] LeTendre ML, Reed MB. The effect of adverse childhood experience on clinical diagnosis of a substance use disorder: Results of a nationally representative study. Substance Use & Misuse. 2017 May 12;52(6):689–97. 28145794 10.1080/10826084.2016.1253746 PMC5519323

[20] U.S. Environmental Protection Agency. Toxicological Review of Tetrachloroethylene (Perchloroethylene). February 2012.

[21] Guyton KZ, Hogan KA, Scott CS, Cooper GS, Bale AS. Human health effects of tetrachloroethylene: key findings and scientific issues. Environmental Health Perspectives. 2014 Apr;122(4):325–34. 24531164 10.1289/ehp.1307359 PMC3984230

[22] National Institute of Alcohol Abuse and Alcoholism. NESARC-III Questionnaire. Section 3C. Medicine Experiences. https://www.niaaa.nih.gov/research/nesarc-iii/questionnaire. Accessed May 20, 2020.

[23] Felitti VJ, Anda RF, Nordenberg D, Williamson DF, Spitz AM. Reprint of: relationship of childhood abuse and household dysfunction to many of the leading causes of death in adults: the adverse childhood experiences (ACE) study. American Journal of Preventive Medicine. 2019 Jun 1;56(6):774–86. Med. 14, 245–258. 31104722 10.1016/j.amepre.2019.04.001

[24] Finkelhor D, Shattuck A, Turner H, Hamby S. Improving the adverse childhood experiences study scale. JAMA Pediatrics. 2013 Jan 1;167(1):70–5. 23403625 10.1001/jamapediatrics.2013.420

[25] Dube SR, Williamson DF, Thompson T, Felitti VJ, Anda RF. Assessing the reliability of retrospective reports of adverse childhood experiences among adult HMO members attending a primary care clinic. Child Abuse & Neglect. 2004 Jul.729–737. 15261468 10.1016/j.chiabu.2003.08.009

[26] Liang KY, Zeger SL. Longitudinal data analysis using generalized linear models. Biometrika. 1986 Apr 1;73(1):13–22.

[27] Zeger SL, Liang KY. Longitudinal data analysis for discrete and continuous outcomes. Biometrics. 1986 Mar 1:121–30. 3719049

[28] Grant BF, Saha TD, Ruan WJ, Goldstein RB, Chou SP. Epidemiology of DSM-5 drug use disorder: Results from the National Epidemiologic Survey on Alcohol and Related Conditions–III. JAMA psychiatry. 2016 Jan 1;73(1):39–47. 26580136 10.1001/jamapsychiatry.2015.2132 PMC5062605

[29] Surzykiewicz J, Skalski SB, Sołbut A, Rutkowski S, Konaszewski K. Resilience and Regulation of Emotions in Adolescents: Serial Mediation Analysis through Self-Esteem and the Perceived Social Support. International Journal of Environmental Research and Public Health. 2022 Jun 29;19(13):8007. 35805666 10.3390/ijerph19138007 PMC9265814

[30] Wingo AP, Wrenn G, Pelletier T, Gutman AR, Bradley B. Moderating effects of resilience on depression in individuals with a history of childhood abuse or trauma exposure. Journal of Affective Disorders. 2010 Nov 1;126(3):411–4. 20488545 10.1016/j.jad.2010.04.009 PMC3606050

[31] Smith C Theorizing religious effects among American adolescents. Journal for the Scientific Study of Religion. 2003 Mar;42(1):17–30.

[32] Liu H, Zhang C, Ji Y, Yang L. Biological and psychological perspectives of resilience: is it possible to improve stress resistance?. Frontiers in Human Neuroscience. 2018 Aug 21;12:326. 30186127 10.3389/fnhum.2018.00326 PMC6110926

[33] Ersche KD, Meng C, Ziauddeen H, Stochl J, Williams GB. Brain networks underlying vulnerability and resilience to drug addiction. Proceedings of the National Academy of Sciences. 2020 Jun 30;117(26):15253–61. 10.1073/pnas.2002509117 PMC7334452 32541059

[34] Martz ME, Zucker RA, Schulenberg JE, Heitzeg MM. Psychosocial and neural indicators of resilience among youth with a family history of substance use disorder. Drug and Alcohol Dependence. 2018 Apr 1;185:198–206. 29462767 10.1016/j.drugalcdep.2017.12.015 PMC5889747

[35] Li Y, Hassett AL, Seng JS. Exploring the mutual regulation between oxytocin and cortisol as a marker of resilience. Archives of Psychiatric Nursing. 2019 Apr 1;33(2):164–73. 30927986 10.1016/j.apnu.2018.11.008 PMC6442937

